# Effects of plyometric training on skill and physical performance in healthy tennis players: A systematic review and meta-analysis

**DOI:** 10.3389/fphys.2022.1024418

**Published:** 2022-11-24

**Authors:** Nuannuan Deng, Kim Geok Soh, Dandan Huang, Borhannudin Abdullah, Shengyao Luo, Watnawat Rattanakoses

**Affiliations:** ^1^ Faculty of Educational Studies, Department of Sports Studies, Universiti Putra Malaysia, Selangor, Malaysia; ^2^ College of Physical Education, Chong Qing University, Chongqing, China; ^3^ Department of Khon Kaen Sport School, Thailand National Sport University, Bueng Nam Rak, Thailand

**Keywords:** plyometric training, skill, speed, power, muscular strength, agility

## Abstract

**Background:** Plyometric training (PT) has been researched extensively in athletic populations. However, the effects of PT on tennis players are less clear.

**Methods:** We aim to consolidate the existing research on the effects of PT on healthy tennis players’ skill and physical performance. On 30th May 2022, a comprehensive search of SCOPUS, PubMed, Web of Science, and SPORTDiscus (*via* EBSCOhost) databases was performed. PICOS was employed to define the inclusion criteria: 1) healthy tennis players; 2) a PT program; 3) compared a plyometric intervention to a control group or another exercise group, and single-group trials; 4) tested at least one measures of tennis skill or physical performance; and 5) non-randomized study trials and randomized control designs. Individual studies’ methodological quality was evaluated by using the Cochrane RoB-2 and ROBINS-I instruments. Using Grading of Recommendations Assessment, Development, and Evaluation (GRADE), the certainty of the body of evidence for each outcome was assessed, and Comprehensive Meta-Analysis software was employed for the meta-analysis.

**Results:** Twelve studies comprising 443 tennis players aged 12.5–25 years were eligible for inclusion. The PT lasted from 3 to 9 weeks. Eight studies provided data to allow for the pooling of results in a meta-analysis. A moderate positive effect was detected for PT programs on maximal serve velocity (ES = 0.75; *p* < 0.0001). In terms of measures of physical performance, small to moderate (ES = 0.43–0.88; *p* = 0.046 to < 0.001) effects were noted for sprint speed, lower extremity muscle power, and agility. While no significant and small effect was noted for lower extremity muscle strength (ES = 0.30; *p* = 0.115). We found no definitive evidence that PT changed other parameters (i.e., serve accuracy, upper extremity power and strength, reaction time, and aerobic endurance). Based on GRADE, the certainty of evidence across the included studies varied from very low to moderate.

**Conclusion:** PT may improve maximal serve velocity and physical performance components (sprint speed, lower extremity muscular power, and agility) for healthy tennis players; however, more high-quality evidence about the effects of PT on the skill and physical performance of tennis players merits further investigation.

**Systematic Review Registration:** [https://inplasy.com/], identifier [INPLASY202250146].

## Introduction

In racket sports such as tennis, technical and tactical skills are crucial for players to reach the top ranks and are primarily related to their performance levels (Strecker et al., 2011; MacCurdy, 2018). Tennis technical skills are most noticeable in serves and groundstrokes (Gillet et al., 2009; Ulbricht et al., 2016; Fauzi et al., 2021). Tactical skills refer to the knowledge of on-court decision-making activities and in-game adaptations (Elferink-Gemser et al., 2010). According to a systematic review, tennis players’ technical and tactical abilities are positively associated with their competition performance levels (Kolman et al., 2019). On the other hand, to be successful in tennis, cardiovascular fitness, sprint rehearsal ability, change of direction speed, muscle strength and muscle power are among the required physical components (Fernandez-Fernandez et al., 2009; Pereira et al., 2016; Björklund et al., 2020). The stronger the physical foundation, the more opportunities for developing technical, tactical, and psychological skills (Bompa and Haff, 2009; Chunlei, 2016; Lambrich and Muehlbauer, 2022), as well as preventing injuries (Carter and Micheli, 2011). Furthermore, it is well acknowledged that players need enhanced levels of physical fitness to execute advanced strokes and compete successfully against more advanced opponents (Ulbricht et al., 2016). Therefore, players require good technical and tactical skills, along with excellent physical performance to succeed in tennis. In this regard, it becomes very important to use training methods specific to the necessities of tennis.

Fortunately, several types of exercise interventions can enhance performance in tennis players. For instance, resistance training can increase serve velocity (Kraemer et al., 2003), while, strength training can enhance forehand and backhand hitting speed (Terraza-Rebollo et al., 2017). Moreover, core training, balance training and sprint training are known to improve the speed and strength of tennis players (Sannicandro et al., 2014; Bashir et al., 2019; Moya-Ramon et al., 2020). High-intensity interval training improves young tennis athletes’ aerobic performance (Fernandez-Fernandez et al., 2017). Notably, among the many kinds of exercises, plyometrics assist to develop power, a foundation from which the athlete can refine the skills of their sport (Davies et al., 2015).

P*lyometric training* involves a stretch-shortening cycle (SSC) that comprises a lengthening action (eccentric movement), followed by a shortening action (concentric movement) (Clark et al., 2016). The mechanism underlying PT mainly involves two parts. The first part transforms the elastic energy stored during muscle stretching into the power output of concentric contraction (Wilk et al., 1993). The second part applies proprioceptor signals induced during muscle stretching to detect muscle tension and length (stretch reflex) (Bal et al., 2012). The sensory signal then sends nerve impulses into the spinal cord to transfer information to alpha motor neurons that activate agonist muscles, recruit motor units, and suppress the contraction of antagonist muscles (Potach, 2004). Meanwhile, the SSC is a model that explains the energy-storing capacities of the series elastic component and the activation of the stretch reflex, which allow for a maximal increase in muscle recruitment in the shortest time (Potach, 2004). There are three distinct phases to the SSC: Prior to muscular activation, phase I, the eccentric phase, increases muscle spindle activity by pre-stretching the muscle; the muscle’s elastic characteristics store the potential energy created during the loading period (Potach, 2004; Davies et al., 2015). Phase II, the amortization phase, is the interval between the conclusion of the eccentric contraction and the beginning of the concentric contraction; during this phase, dynamic stabilization occurs (Cavagna, 1977). Rapid transition from an eccentric contraction to a concentric contraction elicits a strong reaction (Clark et al., 2016). Phase III, the concentric phase, consists of a concentric contraction that improves muscular function after the eccentric phase (Wilk et al., 1993; Clark et al., 2016).

Based on the physiological principles mentioned above, PT optimizes the SSC and related neuro-mechanical mechanisms (Markovic and Mikulic, 2010) has the potential and training advantage in improving sports performance in athletic populations. Previous studies have proven PT’s effects on skill performance, and the results are extremely positive. For instance, Komal and Singh. (2013) indicates that PT of 8 weeks has a significant effect on the dribbling and speed shot performance of basketball players. Hall et al. (2016) found that the implementation of a 6-week PT program can contribute to improving the handspring vault performance of competitive gymnasts. The study by Rubley et al. (2011) found an improvement in the kicking performance of soccer players after a 14-week PT program. At the same time, PT has been shown to be effective in improving athletes’ physical performance (e.g., sprint, jump, muscle strength, balance, endurance, agility, and flexibility) regardless of age, gender, training experience, and competition level (Agostini et al., 2017; Bogdanis et al., 2019; Fathi et al., 2019; Li et al., 2019; Bouteraa et al., 2020; Jlid et al., 2020; Tammam and Hashem, 2020; Ahmadi et al., 2021; Rojano Ortega et al., 2021; Romero et al., 2021; Sáez De Villarreal et al., 2021; Kim et al., 2022; Kosova et al., 2022). Moreover, PT has also been used to benefit the prevention of ankle injuries (Huang et al., 2021). Thus, we formulated two hypotheses based on previous work. First, we hypothesized that PT would improve skill performance in tennis players. Second, we hypothesized PT would exert beneficial effects on physical performance among tennis players.

In recent years, several reviews and meta-analyses on the impact of PT on athletic performance characteristics have been published (Silva et al., 2019; Ramirez-Campillo et al., 2020a; 2020b; 2021a; 2021b). However, these meta-analyses included athletes from various sports (i.e., volleyball players, soccer players, basketball players). Because the effects of PT may differ based on the athlete’s athletic history, the results of these studies cannot be applied directly to tennis players (Ramirez-Campillo et al., 2021b; Sole et al., 2021). According to our knowledge, no previous systematic reviews and meta-analyses have been undertaken on the effects of PT on tennis players, exposing a gap in the literature. Implementing a systematic review and meta-analysis may assist in identifying gaps and limitations in the PT literature and provide practitioners and researchers in adjacent domains with vital information regarding potential future research routes (Ramirez-Campillo et al., 2022). Increasingly, experimental research has investigated the influence of PT on tennis players. For example, comparing PT to conventional tennis exercises, Fernandez-Fernandez et al. (2016) found that PT appears to be an acceptable stimulus for enhancing physical qualities and serve velocity in tennis players. However, this evidence has not yet been gathered comprehensively. Consequently, a systematic review and meta-analysis are required to examine the effects of PT on tennis performance.

Therefore, this systematic review and meta-analysis serves a dual function. The initial purpose of this review was to summarize the present status of the literature on this topic, noting deficiencies in existing research and outlining the path that future researchers should take to address these deficiencies. The second objective was to finally quantify the effects of PT on healthy tennis players in order to determine the influence of this type of training on skill and physical performance, extending the existing understanding of the effects of PT on athletes and widening tennis-specific training methods.

## Methods

There was adherence to the Preferred Reporting Items for Systematic Reviews and Meta-Analyses (PRISMA) guidelines throughout the entire process of conducting this systematic review (Page et al., 2021), and the review protocol has been registered on Inplasy.com (INPLASY202250146).

### Search strategy

We systematically searched SCOPUS, PubMed, Web of Science, and SPORTDiscus (*via* EBSCOhost) electronic databases from inception until 30th May 2022. A systematic investigation of the topic was carried out utilizing the Boolean operations AND and OR. For keyword selection and search strategy development, the authors sought advice from experienced librarians. The keywords are as follows: (“plyometric training” OR “plyometric exercise*” OR “plyometric drill*” OR “plyometr*” OR “ballistic six” OR “ballistic training” OR “explosive” OR “force-velocity” OR “stretch-shortening cycle” OR “stretch-shortening exercise” OR “complex training” OR “jump training”) AND (“tennis” OR “tennis player*” OR “tennis athlete*”). The main databases search string is provided in Supplementary Appendix S1. Furthermore, to find additional literature that might not have shown up in the search results using the four databases, a search was also carried out on Google Scholar and based on the reference lists of selected papers, previously relevant reviews and meta-analyses (Singla et al., 2018; Sole et al., 2021; Colomar et al., 2022; Xiao et al., 2022).

### Study selection

**FIGURE 1:**
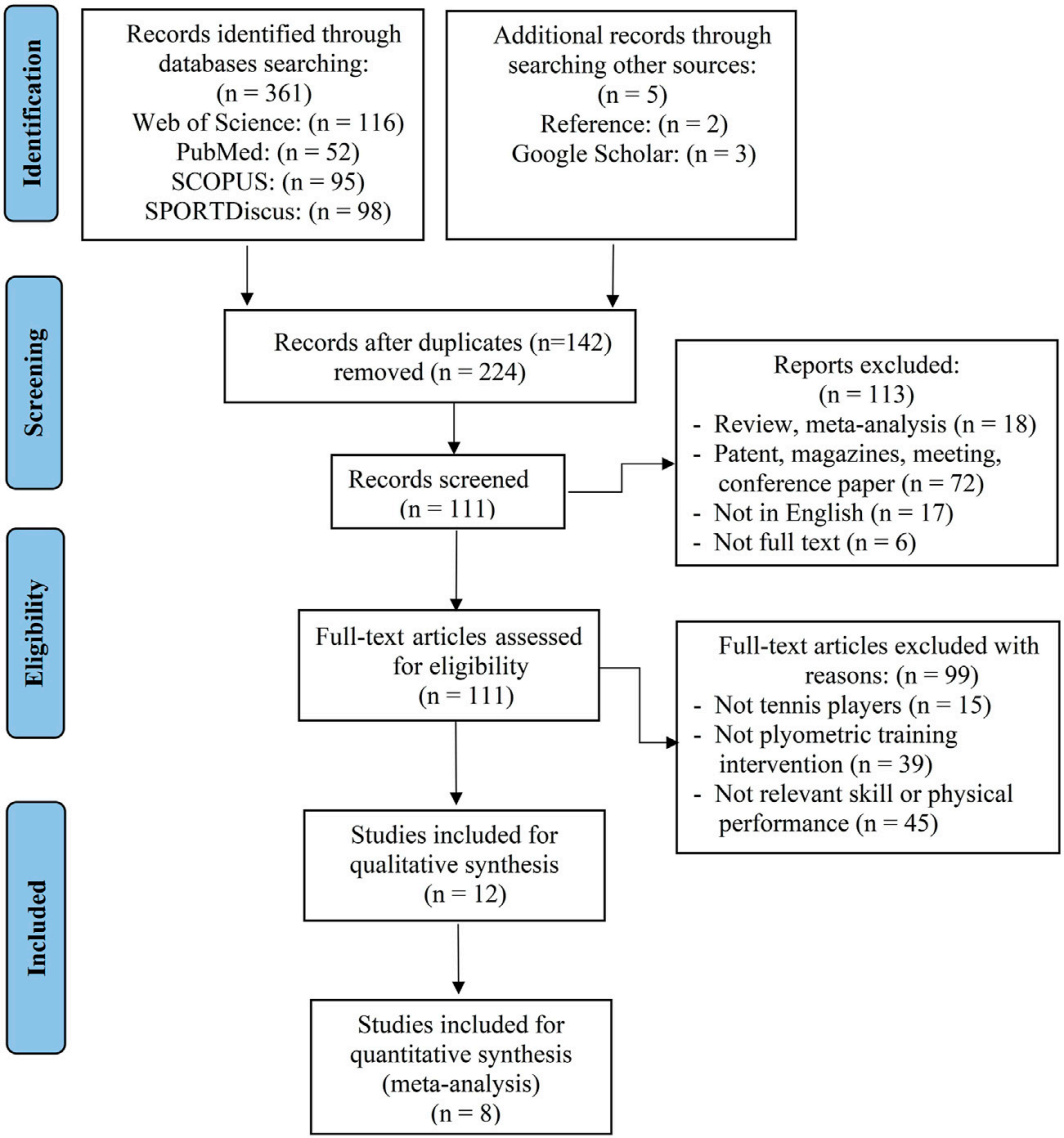
PRISMA flow diagram.

Firstly, two authors (ND, DH) uploaded the collected literature information to the Endnote X9 reference management software based on title, type, authors, and year of publication. After that, all duplicates were removed (ND). Two independent authors (ND, KS) screened each study’s title and abstract for potentially relevant full-text literature. After that, the same authors compared the entire study text to the inclusion and exclusion criteria and chose publications that fulfilled the requirements. Two independent authors (ND, KS) worked out their disagreements through discussion; if they disagreed, a third author (BA) was consulted until they reached an agreement. Figure 1 illustrates the details of the selection procedure.

### Eligibility criteria

To evaluate whether studies could be included in the review, we employed the PICOS approach (Table 1). The selection criteria that we considered for our systematic review are stated below:

1) Healthy tennis players, with no limitations on their gender, age or level.
2) Experimental studies included a PT program to determine tennis players’ skill and/or physical performance; studies using multicomponent training with plyometric components (e.g., neuromuscular training) or combinations of PT with other types of exercise training were also included.
3) Compare a PT intervention to a control or exercise group, and single-group trials were also included. Additionally, studies involving a meta-analysis need to have the mean and standard deviation for the experimental and control groups at pre-and post-test.
4) Reported one or more of the following outcomes: skill performance (e.g., maximal stroke velocity, stroke accuracy) and physical performance (e.g., jump height, sprint speed, agility).
5) To avoid the exclusion of potentially pertinent high-quality research, we took the inclusion of full-text, peer-reviewed, original RCTs and non-RCTs into consideration. Our study focused only on English-language articles, since it may be difficult to translate articles written in different languages, and previous research has demonstrated that nearly all of the literature (99.6%) on plyometric jump training was in English (Ramirez-Campillo et al., 2018).

**TABLE 1:** Eligibility criteria according to the PICOS conditions.

Category	Inclusion criteria	Exclusion criteria
Population	Healthy tennis players	Tennis players with health problems (e.g., injuries, recent surgery)
Intervention	A plyometric training program, defined as upper-body plyometrics (medicine ball exercises, push-ups, and chess press) or lower-body plyometrics (unilateral or bilateral bounds, jumps, hops, and/or skips) or combined upper and lower-body plyometrics that commonly utilize a pre-stretch or countermovement stressing the stretch-shortening cycle	Exercise interventions not involving plyometric training or exercise interventions involving plyometric training programs representing less than 50% of the total training load (i.e., volume, e.g., number of exercises) when delivered in conjunction with other training interventions (e.g., sprint training)
Comparator	Two or more groups and single-group trials	Not under supervision
Outcome	At least one measure related to tennis skill (e.g., maximal stroke velocity, stroke accuracy) or physical performance (e.g., sprint speed, jump height, agility)	Lack of baseline and/or follow-up data
Study design	RCT or non-RCT	Cross-sectional studies, case studies, articles not written in English

RCT, Randomized control trial; Non-RCT, Non-Randomized control trial.

The following exclusion criteria were used in our systematic review:

1) Unhealthy tennis players or those with injuries.
2) Training programs not involving PT or training interventions with plyometric exercises representing less than half of the training volume when delivered with other training interventions (e.g., sprint training).
3) Cross-sectional, review papers and training-related works that did not focus on the impact of PT were excluded.
4) Excluding case reports, brief communications, letters to the editor, invited comments, errata, overtraining studies, patents, and retrospective, papers for which only the abstract was available.

### Data extraction

Two reviewers (ND, DH) retrieved data from each study using a Microsoft Excel spreadsheet (Microsoft Corporation, Redmond, WA, United States), and a third reviewer (KS) validated the data. The data considered were: 1) Name of the first author and year of publication; 2) Subject characteristics: sample size, gender, age (years), and tennis experience; and 3) Characteristics of the PT intervention, which are training protocol, training frequency (days/week), duration (weeks), intensity level (e.g., maximal), rest time between sessions (hours), rest time between sets (s), rest time between repetitions (s), type of progressive PT overload (e.g., volume-based; technique-based), training period (e.g., in-season), PT replaced (if applicable) a component of the regular tennis practice.

### Risk of bias in individual studies and certainty of evidence

According to the guideline on the webpage for Cochrane Training, two reviewers (ND, KS) independently assessed the risk of bias of each of the identified RCTs using the updated Cochrane risk of bias assessment for randomized trials (RoB-2) (Higgins et al., 2022). Risk Of Bias In Non-randomized Research of Interventions (ROBINS-I) was used to evaluate the risk of bias in non-randomized controlled trials (Higgins et al., 2022). GRADE was used to analyze and summarize the confidence of evidence following the GRADE handbook’s principles (Schünemann et al., 2020). First, we categorized all relevant trials based on the outcomes they reported. Then, to determine the confidence of evidence, the following six factors were considered: research design, study limitations, inconsistency, indirectness, imprecision, and publication bias. Outcomes were assessed separately for RCTs and non-RCTs. Two individuals authored this work (ND, KS).

### Data synthesis and meta-analysis

If three or more relatively homogeneous studies supplied explicit pre-and post-test data for the control and experimental groups using the same parameters, these papers were combined for meta-analysis (Ramirez-Campillo et al., 2021b; Ramachandran et al., 2021). In contrast, a narrative synthesis of the findings is conducted (Elgueta-Cancino et al., 2022). On the basis of pre- and post-intervention performance means and standard deviations, between-group effect sizes (ES; Hedge’s g) were computed (SD). The data were standardized using the post-score SD. The inverse-variance random-effects model was utilized for meta-analyses because it assigns a proportional weight to trials depending on the magnitude of their individual standard errors (Deeks et al., 2008) and aids analysis while accounting for heterogeneity across studies (Kontopantelis et al., 2013). If the needed information was not available in the original article or additional materials, the authors were contacted. If contact was not returned or data was unavailable, the study was excluded from the meta-analysis. The ES values were presented with 95% confidence intervals (95% CIs). The ES magnitudes were interpreted using the following scale: < 0.2, trivial; 0.2–0.6, small; > 0.6–1.2, moderate; >1.2–2.0, large; >2.0–4.0, very large; >4.0, extremely large (Hopkins et al., 2009). In some investigations including multiple PT groups, the control group was proportionally divided to permit comparisons across all participants (Higgins et al., 2008). To assess heterogeneity, the I^2^ statistic was employed. Low, moderate, and high degrees of heterogeneity, were determined to be 25%, 25–75%, and >75%, respectively (Higgins et al., 2003). The risk of publication bias across studies was assessed using the extended Egger’s test (Egger et al., 1997). Furthermore, a sensitivity analysis was performed when Egger’s test was significant (*p* < 0.05). An analysis of all available data was carried out using Comprehensive Meta-Analysis software (version three; Biostat, Englewood, NJ, United States).

## Results

### Study selection

The database search produced 361 articles, with an additional five articles discovered *via* Google Scholar and reference lists. In total, 224 research articles remained after duplicates were removed, and the remaining articles were eliminated based on title and abstract screening. An evaluation of the remaining 111 studies was conducted independently by two researchers. The systematic review included twelve studies after the final screening process (Table 2), and eight articles were included in the meta-analysis (Supplementary Appendix S2). The detailed selection procedure for the studies is shown in Figure 1.

**TABLE 2:** Characteristics of the studies examined in the present review.

Study	Design	Population characteristics				Interventions	Comparator	Measures index		Outcomes	
		N	Sex	Age	Exp/level			Skill	Physical	Skill	Physical
Salonikidis and Zafeiridis (2008)	Pre-post test	64	M	21.1 ± 1.3 yrs	2–3 yrs novice	Freq: 3 times/week Time: NR Length: 9 weeks	Plyometric training (EG1), Plyometric **+** tennis regular drills (EG2), Control group (CG)		Reaction time (RTSS); Sprint (4 mSS, 4 mFS, 12 mFS, 12 mFST); Strength (L: Fmax); Power (L: DJ)		EG1:RTSS↑, 4mSS↑, 4mFS↑, 12mFS↔, 12mFST↔, DJ↑, Fmax↑; EG2: RT↑, 4mSS↑, 4mFS↑, 12mFS↑, 12mFST↑, DJ↑, Fmax↑
Gelen et al. (2012)	within-subject, repeated-measures	26	NR	15.1 ± 4.2 yrs	8.4 ± 3.8 yrs elite	Time: 34 min	Static stretching (EG1), Dynamic exercises (EG2), Plyometric exercises (EG3), Control group (CG)	Serve (SV)		SV↑	
Behringer et al. (2013)	Pre-post test	36	M	15.03 ± 1.64 yrs	averaged 6.15 yrs local tennis club	Freq: 2 times/week Time: 45 min Length: 8 weeks	Resistance training (EG1), Plyometric training (EG2), Control group (CG)	Serve (SV, SA)	Strength (10RM test: U: chest press, pull-down machine, abdominal press; L: leg press)	SV↑, SA↔	10RM test↑
Ölçücü et al. (2013)	Pre-post test	40	M	20–25 yrs	NR tournament	Freq: 2 times/week Time: 35 min Length: 8 weeks	Plyometric training (EG), Control group (CG)	Serve (SV)	Strength (isokinetic test, U: shoulder joint; L: knee joint)	SV↑	All isokinetic tests ↑
Fernandez-Fernandez et al. (2015)	Pre-post test	16	M	16.9 ± 0.5 yrs	8.0 ± 2.6 yrs elite	Freq: 2 times/week Time: 30–60 min Length: 8 weeks	Explosive strength and repeated sprint training (EG), Control group (CG)		Sprint (10 m, 20 m, 30 m, RSAb, RSAm); Power (L: CMJ); Aerobic endurance (VIFT)		10 m↑, CMJ↑, RSAb↑, RSAm↑, 20 m↔, 30 m↔, VIFT↔
Fernandez-Fernandez et al. (2016)	Pre-post test	60	M	12.5 ± 0.3 yrs	>3 yrs international tennis academy	Freq: 2 times/week Time: 30–60 min Length: 8 weeks	Plyometric training (EG), Control group (CG)	Serve (SV, SA)	Sprint (5 m,10 m,20 m); Power (U: MBT; L: CMJ, SLJ); Agility (5-0–5 test)	SV↑, SA↑	5 m↑, 10 m↑, 20 m↑, 5-0–5 test↑, SLJ↑, MBT↑, CMJ↑
Rathore (2016)	Pre-post test	60	M	18–23 yrs	NR professional	Freq: 3 times/week Time: 45 min Length: 8 weeks	Plyometric training (EG1), Resistance training (EG2), Control group (CG)		Agility (Illinois test)		Illinois test↑
Fernandez-Fernandez et al. (2018)	Pre-post test	16	M	12.9 ± 0.4 yrs	3.0 ± 1.2 yrs elite	Freq: 2 times/week Time: 20–40 min Length: 8 weeks	NMT before tennis-specific training (EG1), NMT after tennis-specific training (EG2)	Serve (SV)	Sprint (5, 10, and 20 m); Power (U: MBT; L: CMJ); Agility (5-0-5 test)	SV→	EG1: 5 m↑, 10 m↑, 20 m↑, 5-0-5test↑, CMJ↑, MBT↑, SV↑; EG2: 10 m↑, 20 m↓, 5 m↓, 5–05-test↓, MBT↔, CMJ↓
Lakshmikanth et al. (2018)	Pre-post test	30	Mixed	18–22 yrs	NR college students	Freq: 1 times/week Time: NR Length: 6 weeks	Plyometric training (EG1), Control group (CG)		Agility (Illinois test, tennis-specific agility test)		Illinois test↑, tennis-specific agility test↑
Ziagkas et al. (2019)	Pre-post test	24	M	20.9 ± 0.66 yrs	1–3 yrs amateur	Freq: 2 times/week Time:30–60 min Length:8 weeks	Plyometric training (EG), Control group (CG)		Agility (Hexagon Test, Spider Test)		Hexagon Test↑, Spider Test↑
Mohanta et al. (2019)	Pre-post test	40	M	18–25 yrs	>2 yrs local tennis academy	Freq: 2 times/week Time: 30–60 min Length: 8 weeks	Plyometric training (EG1), Circuit training (EG2)		Sprint (50-meter dash test); Strength (U:1RM chest press test); Power (L: VJ); Agility (T-test)		50-meter dash test↑, 1RM chest press test↑, VJ↑, T-test↑
Hotwani et al. (2021)	Pre-post test	31	Mixed	15–18 yrs	NR local tennis academy	Freq: NR Time: 60 min Length: 3 weeks	Plyometrics + sprint training (EG)		Agility (T-test, Illinois Test)		T-test↑, Illinois Test↑

NR, Not reported; yrs, Years; Exp, Tennis experience; M, Male; F, Female; Freq, Frequency; CG, Control group; EG, Experimental group; NMT, Neuromuscular training; CMJ, Vertical countermovement jump; SLJ, Standing long jump; 505 Test, modified 505 agility test; DJ, drop jump; VJ, Vertical jump; MBT, Medicine ball throw; SV, Serve velocity; SA, Serve accuracy; RTSS, Reaction time single step; 4mSS, 4-m side-step; 4mFS, 4-m forward sprint; 12 mFS, 12-m forward sprint; 12 mFST, 12-m forward sprint with turn; RSAb, Repeated sprint ability best; RSAm, Repeated sprint ability mean; 10RM, 10 Repetition Maximum Testing; VIFT, Velocity of the intermittent fitness test; 1RM, 1 Repetition maximum; 10RM; 10 Repetition maximum; Fmax, Maximum isometric force (leg); U, Upper extremity; L, Lower extremity; ↑, significant within-group improvement; ↔, non-significant within-group.

### Risk of bias in individual studies and certainty of evidence

**FIGURE 2:**
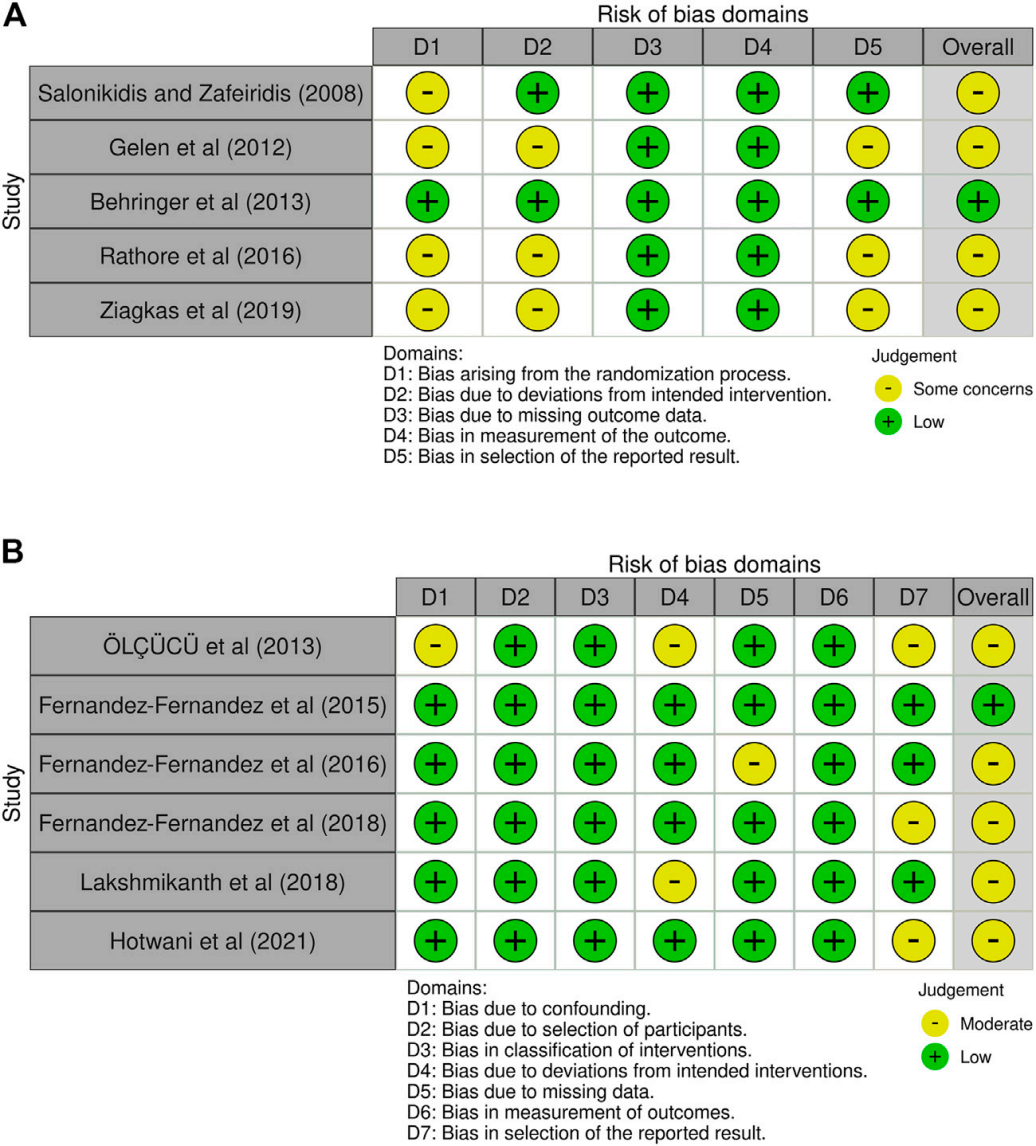
Risk of bias. **(A)** Results for RCTs, **(B)** results for non-RCTs. *Created using Robvis (visualization tool): McGuinness and Higgins (2021).

RoB-2 was applied to five RCTs, while ROBINS-I was applied to seven non-RCTs. Ten trials were found to have an overall moderate risk of bias or presented some concerns, only two papers showed a lower risk of bias (see Figure 2). Figure 2A depicts the findings of the RoB-2 evaluations. Only one research described the method for generating randomization sequences utilizing stratified block randomization and specifically documented allocation concealment (Behringer et al., 2013), whereas four articles did not completely describe the randomized procedure (Salonikidis and Zafeiridis, 2008; Gelen et al., 2012; Rathore, 2016; Ziagkas et al., 2019). Only two studies had preregistered protocols (Salonikidis and Zafeiridis, 2008; Behringer et al., 2013), and three of the studies therefore had some concerns regarding bias in the selection of the reported results (Gelen et al., 2012; Rathore, 2016; Ziagkas et al., 2019). Figure 2B depicts a graphical representation of the outcomes of ROBINS-I evaluations. One of the non-RCTs discovered some concerns due to missing data, owing to an approximate 15% dropout rate (Fernandez-Fernandez et al., 2016), two studies had moderate risk of bias in domain of deviations from intended intervention (Ölçücü, 2013; Lakshmikanth et al., 2018), and three studies had moderate risk regarding bias in the selection of the reported results (Ölçücü et al., 2013; Fernandez-Fernandez et al., 2018; Hotwani et al., 2021).

According to the GRADE assessment (Supplementary Appendix S3), in terms of RCTs, the certainty of evidence is considered very lower to moderate. In terms of non-RCTs, the certainty of evidence is considered very lower to low.

### Study characteristics

Table 2 summarizes the study and intervention characteristics. Moreover, the training protocol of each study can be found in Supplementary Appendix S4. The twelve included papers were carried out between 2008 and 2021, with 443 tennis players. Across the studies, 1) Gender: nine studies focused on men (Salonikidis and Zafeiridis, 2008; Behringer et al., 2013; Fernandez-Fernandez et al., 2015; Fernandez-Fernandez et al., 2016; Fernandez-Fernandez et al., 2018; Mohanta et al., 2019), no studies focused on female tennis players alone, two studies reported mixed gender (Lakshmikanth et al., 2018; Hotwani et al., 2021), and one study did not specify gender (Gelen et al., 2012); 2) Age: all studies recorded the participants’ ages, and an overview of age reports from twelve research revealed that the participants’ ages ranged from 12.5 to 25 years; 3) Tennis experience: eight studies reported on the training experience of the tennis players (Salonikidis and Zafeiridis, 2008; Gelen et al., 2012; Behringer et al., 2013; Fernandez-Fernandez et al., 2015; Fernandez-Fernandez et al., 2016; Fernandez-Fernandez et al.2018; Mohanta et al., 2019), ranging from 12 to 96 months, four studies did not report on training experience (Ölçücü et al., 2013; Rathore, 2016; Lakshmikanth et al., 2018; Hotwani et al., 2021); 4) Intervention: regarding the training regimen in this review, four studies conducted PT for the upper and lower extremities (Behringer et al., 2013; Fernandez-Fernandez et al., 2016; Mohanta et al., 2019; Ziagkas et al., 2019), and one study used only upper limb PT (Gelen et al., 2012), six studies used lower-extremity PT (Salonikidis and Zafeiridis, 2008; Fernandez-Fernandez et al., 2015; Rathore, 2016; Fernandez-Fernandez et al., 2018; Lakshmikanth et al., 2018; Hotwani et al., 2021), but three studies did not detailed description the training protocol (Ölçücü, 2013; Rathore, 2016; Ziagkas et al., 2019). In addition, three studies combined PT with other types of exercise training (i.e., sprint training, acceleration/deceleration/change of direction drills) (Fernandez-Fernandez et al., 2015; Fernandez-Fernandez et al., 2018; Hotwani et al., 2021). The frequency of training was one to three times per week, and two studies did not report the frequency (Gelen et al., 2012; Hotwani et al., 2021). The duration of the intervention was primarily between 20 and 60 min, but one study did not report the period (Salonikidis and Zafeiridis, 2008). The interventions ranged from three to 9 weeks, and most of the interventions were conducted for 8 weeks (*n* = 8), and only one study did not report the length (Gelen et al., 2012).

### Results from meta-analysis

The meta-analysis was conducted on certain trials that measured maximal serve velocity (*n* = 3), sprint speed (*n* = 3), lower extremity power (*n* = 3) and strength (*n* = 3), agility (*n* = 4). Other outcomes were insufficient and produced limited data for pooling, consequently, they were not considered for meta-analysis. The data used for meta-analyses and the forest plots are presented in Supplementary Appendix S3, S5 **,** respectively.

The PT showed a significant increase (*p* < 0.001) of maximal serve velocity with moderate effect (ES = 0.75; 95% CI = 0.38–1.12; Egger’s test *p* = 1.00; *n* = 115) and low heterogeneity (I^2^ = 0.0%) (Supplementary Appendix S2; Supplementary Figure S1).

The PT showed a significant increase (*p* = 0.046) of sprint speed with moderate effect (ES = 0.43; 95% CI = 0.01–0.85; Egger’s test *p* = 1.00; *n* = 115) and low heterogeneity (I^2^ = 18.7%) (Supplementary Appendix S2; Supplementary Figure S2).

The PT showed a significant increase (*p* = 0.022) of lower extremity power with small effect (ES = 0.50; 95% CI = 0.07–0.93; Egger’s test *p* = 0.734; *n* = 115) and lower heterogeneity (I^2^ = 19.9%) (Supplementary Appendix S2; Supplementary Figure S3).

The PT showed a non-significant change (*p* = 0.115) in lower extremity muscle strength with small effect (ES = 0.30; 95% CI =-0.07–0.68; Egger’s test *p* = 0.471; *n* = 108) and lower heterogeneity (I^2^ = 0.0%) (Supplementary Appendix S2; Supplementary Figure S4).

The PT showed a significant increase (*p* < 0.001) of agility with moderate effect (ES = 0.88; 95% CI = 0.53–1.23; Egger’s test *p* = 0.060; *n* = 105) and lower heterogeneity (I^2^ = 6.42%) (Supplementary Appendix S2; Supplementary Figure S5).

## Synthesis of results

### Effect of plyometric training on maximal serve velocity and accuracy

Five studies contained in this review evaluated maximal serve velocity. Among these studies, ball speed measurement by radar gun (Gelen et al., 2012; Behringer et al., 2013; Ölçücü et al., 2013; Fernandez-Fernandez et al., 2016; Fernandez-Fernandez et al., 2018). Two RCTs measured this skill aspect. Gelen et al. (2012) compared EG3 and CG employing high-volume upper body PT *vs*. normal tennis practice. They discovered a significant (*p* = 0.001) acute effect in maximal serve speed. Behringer et al. (2013) conducted an 8-week PT for the upper and lower bodies and increased the maximum serve velocity in a statistically significant (*p* < 0.05) way. Three non-RCTs studied maximal serve velocity. Fernandez-Fernandez et al. (2016) found a significant increase (*p* < 0.05) in maximal serve velocity after an 8-week upper and lower body PT program. Meanwhile, in another study, the comparison of PT *versus* a traditional training regimen indicated positive effects on maximal serve velocity (*p* < 0.01) (Ölçücü et al., 2013). In addition, the report by Fernandez-Fernandez et al. (2018) demonstrates that the EG1 significantly improved the maximal serve velocity. In contrast, the EG2 did not observe any changes.

One RCT (Behringer et al., 2013) and one non-RCT (Fernandez-Fernandez et al., 2016) measured serve accuracy. The accuracy of the service is scored by calculating the position of the ball landing in the specified target area. The RCT reported neither positive nor adverse impacts have been observed on service accuracy scores after an 8-week PT program (Behringer et al., 2013). Interestingly, Fernandez-Fernandez et al. (2016) reported a significant change (*p* < 0.01) in serve accuracy after a similar 8-week PT program.

### Effect of plyometric training on sprint speed

Five of the studies (one RCT and four non-RCTs) assessed sprint speed. The sprint tests used in these investigations comprised the linear sprint test of 4 m (Salonikidis and Zafeiridis, 2008), 5 m (Fernandez-Fernandez et al., 2016), 10 m (Fernandez-Fernandez et al., 2015; Fernandez-Fernandez et al., 2016; Fernandez-Fernandez et al., 2018), 12 m (Salonikidis and Zafeiridis, 2008), 20 m (Fernandez-Fernandez et al., 2015; Fernandez-Fernandez et al., 2016; Fernandez-Fernandez et al., 2018), 30 m (Fernandez-Fernandez et al., 2015), 50 m (Mohanta et al., 2019); lateral sprint test of 4 m (Salonikidis and Zafeiridis, 2008); and change of direction sprint test of 12 m with turn (Salonikidis and Zafeiridis, 2008), repeated sprint ability (Fernandez-Fernandez et al., 2015).

The RCT reveals that it significantly improves (*p* < 0.05) 12 m sprint without turn performance in the combined group (PT + regular tennis drills) and PT alone group. Meanwhile, the performance in the 12 m sprint with turn was improved only in the EG2 and failed to reach significant differences in the EG1 (Salonikidis and Zafeiridis. 2008). Three 8-week non-RCTs revealed improvements (*p* < 0.05) in the linear sprint test (Fernandez-Fernandez et al., 2016; Mohanta et al., 2019) and repeated sprint ability test (Fernandez-Fernandez et al., 2015). Of note, Fernandez-Fernandez et al. (2015) reported that the 10 m test was significantly improved (*p* < 0.05), in contrast to the 20 m test and 30 m test. Fernandez-Fernandez et al. (2018) reported that the EG1 had a positive effect on pre-to post-test measures of the sprint, while the EG2 training method had negative effects on 5 m, or trivial effects on 10 m and 20 m.

### Effect of plyometric training on upper and lower extremity power

Two non-RCTs measured upper extremity power *via* medicine ball throw tests (Fernandez-Fernandez et al., 2016; Fernandez-Fernandez et al., 2018). Fernandez-Fernandez et al. (2016) compared EG and CG applying an 8-week PT program *vs*. normal tennis practice. They discovered that MBT performance had improved significantly (*p* < 0.05). Fernandez-Fernandez et al., 2018 observed a considerable increase in MBT following the EG1 training method, but no improvement in EG2.

One RCT (Salonikidis and Zafeiridis, 2008) and four non-RCTs (Fernandez-Fernandez et al., 2015; Fernandez-Fernandez et al., 2016; Fernandez-Fernandez et al., 2018; Mohanta et al., 2019) measured lower extremity power. This outcome test consisted of CMJ test (Fernandez-Fernandez et al., 2015; Fernandez-Fernandez et al., 2016; Fernandez-Fernandez et al., 2018), drop jump test (Salonikidis and Zafeiridis, 2008), SLJ test (Fernandez-Fernandez et al., 2016), VJ test (Mohanta et al., 2019). Salonikidis and Zafeiridis (2008) found that EG2 activities had a significant gain (*p* < 0.05) in DJ height when compared to EG1. Fernandez-Fernandez et al. (2016) demonstrate that EG *vs*. CG significantly enhanced (*p* < 0.01) CMJ and SLJ performance. Fernandez-Fernandez et al. (2015) compared EG and CG using an 8-week combination of explosive strength and repeated sprint training. The results indicate that CMJ achieved a positive effect. Fernandez-Fernandez et al. (2018) reported data on EG1 *vs*. EG2, and it is noted that the EG1 training approach markedly improved (*p* < 0.05) the CMJ performance, in contrast to the EG2. Furthermore, Mohanta et al. (2019) showed that 8 weeks of PT could significantly improve VJ height (*p* = 0.027).

### Effect of plyometric training on upper and lower extremity strength

One RCT (Behringer et al., 2013) and two non-RCTs (Ölçücü et al., 2013; Mohanta et al., 2019) measured upper extremity strength. The tests applied at this point involved the 1RM and 10RM chest press test (Behringer et al., 2013; Mohanta et al., 2019), and the torque of the shoulder joint test (Ölçücü et al., 2013). The RCT (8-week PT program) conducted by Behringer et al. (2013) observed a greater increase (*p* < 0.05) in upper extremity strength (10RM chest press test) when compared to the control group. Two similar 8-week PT programs (non-RCT design) also found a statistically significant difference (*p* < 0.05) in upper limb strength (1RM chest press and torque of the shoulder joint test) measurement between the experimental group’s pre-and post-test (Ölçücü et al., 2013; Mohanta et al., 2019).

The lower extremity strength test consists of a 10RM leg press test (Behringer et al., 2013), a maximum isometric force test (leg) (Salonikidis and Zafeiridis, 2008), and a torque of the knee joint test (Ölçücü et al., 2013). Two RCTs indicated that tennis players who trained with 8 weeks of PT had considerably larger increases in lower extremity strength (*p* < 0.05) than the control group (Salonikidis and Zafeiridis, 2008; Behringer et al., 2013). One non-RCT also achieved a positive effect in the strength test (Ölçücü et al., 2013) after 8-week PT.

### Effect of plyometric training on agility

In the twelve studies that were reviewed, agility was reported in seven. The factors valued and assessment instruments used included the 5-0-5 test (Fernandez-Fernandez et al., 2016; Fernandez-Fernandez et al., 2018), T-test (Mohanta et al., 2019; Hotwani et al., 2021), Illinois test (Rathore, 2016; Lakshmikanth et al., 2018; Hotwani et al., 2021), tennis-specific agility test (Lakshmikanth et al., 2018), hexagon test and spider test (Ziagkas et al., 2019). Two RCTs (Rathore, 2016; Ziagkas et al., 2019) show that EG *vs*. CG substantially increased agility performance (hexagon test, spider test, Illinois test; *p* < 0.0001). Among non-RCTs, a statistically significant difference between the pre-and post-test in several agility tests (i.e., 5-0-5 test, T-test, Illinois test, tennis-specific agility test) was revealed by the EG of studies (Fernandez-Fernandez et al., 2016; Lakshmikanth et al., 2018; Mohanta et al., 2019). Moreover, Hotwani et al., 2021 revealed that 3 weeks of combining plyometric with sprint training sessions resulted in an extremely significant (*p* < 0.0001) gain in agility (Illinois test and T-test). However, Fernandez-Fernandez et al. (2018) discovered positive effects from pre-test to post-test measures on agility (5-0-5 test) in the EG1. On the contrary, the agility test for the EG2 showed negative impacts.

### Effect of plyometric training on reaction time/aerobic endurance

One RCT (Salonikidis and Zafeiridis, 2008) and one non-RCT (Fernandez-Fernandez et al., 2015) evaluated reaction time and aerobic endurance performance, respectively.

The reaction time was measured based on the load cells. Salonikidis and Zafeiridis 2008 reported outcomes on EG1 *vs*. EG2; both PT regimens induce favorable changes to reaction time performance (*p* < 0.05). Another study using the 30–15 interval fitness test evaluated aerobic endurance performance, and the study reported no significant improvement in this aspect of factor (Fernandez-Fernandez et al., 2015).

### Adverse effects

It is worthy to note that one out of the twelve studies reported that eight players in the control group and one in the PT group were dropped from the final collection of data because of acute injuries (i.e., ankle sprain) (Fernandez-Fernandez et al., 2016). Apart from that, no other studies in this review reported fatigue, soreness, injury, pain, damage, or adverse effects associated with PT intervention.

## Discussion

This is the first meta-analysis to investigate the impact of PT on healthy tennis players. This study involved twelve trials, and five studies involved skill, but only serve performance. Eleven articles were related to physical performance. Besides, no data were found on body composition, balance, flexibility, and other aspects of skills (Table 2). The results demonstrated that PT interventions induced small-to-moderate improvements (ES = 0.43–0.88) in tennis players’ maximal serve velocity and physical performance parameters (i.e., sprint speed, lower body power, agility), and a non-significant, small improvement (ES = 0.30) in lower extremity strength. In most cases, the results indicated above had a low level of heterogeneity (I^2^ = 0.0%–19.9%). In this review, one study reported acute injuries during the intervention which the authors had recorded. It is still necessary that coaches and players remain cautious and vigilant about injuries sustained during PT drills, and that the training must be performed under supervision. Furthermore, only two of the twelve studies in this work were rated with a low risk of bias. Additionally, a very low to moderate level of evidence was reported for the measured variables. Therefore, the outcomes should be interpreted with caution.

### Effect of plyometric training on maximal serve velocity and accuracy

The tennis serve is undoubtedly one of the most challenging tennis shots to master, but it can significantly contribute to a point win or advantage (Guillot et al., 2013). Based on our meta-analysis, PT interventions were found to have a moderate effect (ES = 0.75) on maximal serve velocity in tennis players. The serve is a complicated stroke that involves a sequence of forces (legs, trunk, and arm/racquet) moving from proximal to distal (Elliott, 2006). During a serve, it is crucial to transfer power from the legs, trunk, and arm to the ball as quickly as possible to maximize the velocity (van den Tillaar. 2004). According to Ferrauti and Bastiaens, (2007), service velocity results from an efficient force transfer along a convoluted kinetic chain dependent on intermuscular coordination and muscle strength. Simultaneously, PT helps to increase muscular strength and intermuscular coordination, so that force transfer during service can be improved (Fernandez-Fernandez et al., 2016). Previously, resistance training has been recommended to increase ball velocity in tennis (Kraemer et al., 2000; Kraemer et al., 2003); yet, a study in the current review specifically compared the effects of PT and resistance training on this factor and found that serve speed improvement was significantly higher in the PT group than in the resistance training group (Behringer et al., 2013). In addition, Fernandez-Fernandez et al., 2013 suggested that upper-extremity plyometric exercises can be frequently used by athletes in the pursuit of more powerful functional performance. In the present review, of the five studies measuring serve speed, four designed upper-body plyometrics in the training protocols (Supplementary Appendix S4), therefore, the increase in maximal serve velocity was expected.

In tennis, the main determinants of shot quality include not only ball velocity but also ball placement accuracy (Kovacs and Ellenbecker, 2011). Fernandez-Fernandez et al. (2016) speculated that the improved kinetic chain as a result of the power gains from the PT would contribute to the stability of tennis players from a technical perspective, leading to a higher accuracy test score. However, another study found that similar plyometric exercises did not affect serve accuracy (Behringer et al., 2013). In the literature, service accuracy was also unaffected by other forms of strength training (Ferrauti and Bastiaens, 2007; Fernandez-Fernandez et al., 2013). Terraza-Rebollo and Baiget. (2020) explained in their study that it was likely because subjects were instructed to hit the ball at maximum speed, so it was difficult to take into account the accuracy. However, there is some controversy because it has also been observed a positive correlation between velocity and accuracy in tennis (Cauraugh et al., 1990).

Therefore, PT is an effective method that may have greater chances of improvement in tennis players’ maximal serve velocity. Concerning serve accuracy, it was impossible to reach a firm conclusion, because only two studies examined the effect of PT on this point. Furthermore, more research is required to explore the relationship between training-induced increases in serve velocity and accuracy.

### Effect of plyometric training on sprint speed

Better sprint performance will enable tennis players to get to the ball faster and will give them more time to prepare for the shot (Kramer et al., 2021). The meta-analysis, summarizing the effects of PT on the sprint speed performance of tennis players, showed a small but significant effect across studies (ES = 0.43). Increases in sprint performance following PT may be due to increased neuromuscular activation of the exercised muscles (Hakkinen and Komi, 1985). Moreover, these adaptations induced by lower body plyometric exercises (e.g., sprinting, hopping, jumping), such as improving muscle-tendon stiffness and increasing neural drive to agonist muscles (Markovic and Mikulic, 2010) may improve SSC efficacy. As a result of gains in SSC efficacy in lower limb musculature, stronger force generation likely occurs in the concentric action phase after a rapid eccentric muscle movement (Markovic and Mikulic., 2010; Radnor et al., 2018), which is a key requirement for enhancing tennis players’ sprint performance. However, one study found that PT had no effect on a 12 m sprint with a turn test (Salonikidis and Zafeiridis, 2008). This observation may have resulted from the activity being conducted only on one leg (Salonikidis and Zafeiridis, 2008). Furthermore, Fernandez-Fernandez et al. (2015) concluded that a PT program did not result in significant gains in the 20 m and 30 m sprint measurements. This discovery might be connected to the athletes’ stride frequency and the coordination of the lower limb muscles (Young et al., 2001). In fact, only a few studies in the literature did not report the positive effects of PT on sprint performance (Oxfeldt et al., 2019). For example, Hammami et al. (2019) investigated the impact of a night-week PT program on young female handball players. There were no changes reported for the 5 m and 10 m sprint times, which could be explained by the fact that initial acceleration (over 5 and 10 m) has proven to be more difficult to improve than maximal velocity, most likely due to the smaller margin for improvement and the different forces involved. Interestingly, Hammami et al. (2020) found inconsistent results on young female handball players, that there were significant increases in speed over distances of 5–30 m after 10 weeks of similar plyometric exercises. Differences between PT programs (e.g., frequency, duration) may help to explain the different magnitudes of physical fitness changes among studies (Ramirez-Campillo et al., 2020b). However, the paucity of available data limits our attempts to explore the effects of these factors on training effects. Therefore, although we have found evidence to support the use of PT as an effective training modality to improve sprint performance, more high-quality research on sprint speed performance is still needed.

### Effect of plyometric training on upper and lower extremity muscle power

Power development is paramount, irrespective of the sport and proportion of each energy system engaged, since certain critical actions are executed as quickly and forcefully as possible (Elliott et al., 2007). On the one hand, upper extremity power was studied in two studies included in this review, and this variable was examined using medicine ball throws. These outcomes showed positive effects (Fernandez-Fernandez et al., 2016; Fernandez-Fernandez et al., 2018). Ulbricht and others discovered that the upper extremity power test (MBT) was an important predictor of tennis performance (Ulbricht et al., 2016). These two studies in our review incorporating PT of the upper limb involving multiple medicine ball exercises could be an important factor in improving upper extremity power. Furthermore, PT works by utilizing the natural elastic components of muscles and tendons, and stretch reflexes, to increase the power of subsequent movements (Trajkovic et al., 2016).

On the other hand, a meta-analysis of lower extremity power (CMJ) was undertaken, and the PT indicated a significant increase with a small effect (ES = 0.50). Such effects were noted for participants with a wide range of sports backgrounds. A meta-analysis on individual sports athletes was done by Sole et al. (2021), and the results showed similar improvements in the CMJ performance of ES = 0.49. In short, factors including improved motor unit recruitment, increased intermuscular coordination, increased neural drive to the muscles of the agonist, and improved SSC utilization are responsible for jump performance improvements (Markovic and Mikulic, 2010; Taube et al., 2012). Of note, a study conducted by Fernandez-Fernandez et al. (2018) showed that performing NMT before regular tennis practice produced a substantially significant positive effect on CMJ. Conversely, when NMT was conducted after regular tennis drills, there was no improvement in CMJ assessed. The previous tennis regular practice that resulted in acute fatigue may have contributed to the lack of a marked increase in CMJ (Garcia-Pallares and Izquierdo, 2011). From this, the optimal design and implementation of training strategies are significant for tennis players. This should be taken into account in future research.

Overall, our findings imply that PT positively affects tennis players’ lower extremity power, and future research should consider further research on upper extremity power.

### Effect of plyometric training on upper and lower extremity muscle strength

For successful athletic performance, strength is essential (Slimani et al., 2016). Moreover, it has been reported that a tennis player’s upper and lower body strength can be very helpful in preventing injuries (Ellenbecker and Roetert, 2004). Of the four studies included in this review assessing strength, three reported upper body strength. These studies showed significant improvement in upper extremity strength tests (Ölçücü et al., 2013, Behringer et al., 2013; Mohanta et al., 2019).

Meanwhile, it has been found in the present meta-analysis that PT led to small gains in lower extremity muscle strength (ES = 0.30), but this was not statistically significant. In contrast, de Villarreal et al. (2010) observed that PT has a significant, moderate effect on lower limb muscle strength (ES = 0.97). Theoretically, PT increases cross-bridge mechanics, activates motor units, enhances neural efficiency, and provides passive tension to the muscle-tendon complex, contributing to strength performance (Malisoux, 2005; Ramírez-Campillo et al., 2015). Moreover, improvements in muscle strength after PT may also be associated with muscle hypertrophy (Grgic et al., 2020).

Additionally, the narrative synthesis in our review showed a positive direction of evidence for muscle strength, but the certainty of the evidence was very low to low. As a result, these findings are unclear, though this could be due to the relatively few studies conducted in this field. Likewise, although our meta-analysis results do not show a significant effect from PT on lower extremity muscle strength, numerous previous studies have concluded that PT is an efficient training technique to enhance strength performance in other ball players, such as basketball players (Ramirez-Campillo et al., 2021a), handball players (Hammami et al., 2020). Therefore, to confirm the effects of PT on tennis players, further high-quality studies simultaneously evaluating the influence of upper and lower body muscle strength are needed to draw more solid conclusions.

### Effect of plyometric training on agility

The basic requirement for a tennis player is the ability to quickly switch between multidirectional movements (e.g., vertical, lateral, forward, backward) (Rathore, 2016). In the present review, one of the most studied physical attributes is agility (seven papers). The findings in the current meta-analysis show that PT has a significant, moderate effect (ES = 0.88) on agility. Similar improvement in agility performance was observed by Asadi et al. (2016) and Thapa et al. (2021), supporting the findings of our study. The present research evaluated agility performance using a variety of tests (5-0–5 test, T-test, Illinois test, Heroxge test, tennis-specific agility test). Of note, Asadi et al. (2016) suggested that PT could improve change-of-direction ability (ES = 0.26–2.8, small-to-large), depending on the types of plyometric exercises and change-of-direction tests. The current results are consonant with these findings, with extensively positive results in all of the agility tests after PT.

Additionally, Fernandez-Fernandez et al. (2018) found positive effects from pre-test to post-test analyzes on agility in the EG1. In contrast, the EG2 observed negative effects on agility. As aforementioned, the fatigue of regular tennis training may have contributed to the decline in agility. In the literature, it has been well identified that PT is a time-efficient, effective and simple method for improving agility among athletes (Asadi, 2013; Arazi et al., 2014; Ramírez-Campillo et al., 2014). Indeed, PT helps reduce ground contact by boosting muscular force output and movement efficiency, which is related to agility improvement (Markovic and Mikulic, 2010; Asadi et al., 2016). Moreover, this type of training approach could have increased eccentric strength in the legs, allowing athletes better to switch between deceleration and acceleration movements (Brughelli et al., 2008). Meanwhile, plyometric exercises which included powerful multidirectional movements helped improve the ability to change directions rapidly (Söhnlein et al., 2014). Therefore, the findings suggest that PT might assist tennis players to enhance their agility performance.

### Effect of plyometric training on reaction time/aerobic endurance

The tennis player’s ability to react quickly in response to their competitor’s movements is crucial during a game (Reid et al., 2013). Reaction time performance has only been investigated in one study, and this study reported significant improvement in the reaction time test (Salonikidis and Zafeiridis, 2008). Similar results were found in a study done by Turgut et al. (2019) which showed a significant improvement in reaction time as a result of PT. However, discussion on the mechanisms related to improved reaction time in athletes after PT remains unclear, with extensive empirical research required to elucidate such mechanisms.

Likewise, aerobic endurance is a major component of physical fitness (Wezenberg et al., 2013), and can be considered one of the benefits of PT. However, only one paper examined aerobic endurance in the current review, and this study found no significant improvement in the aerobic endurance test (30–15 interval fitness test) (Fernandez-Fernandez et al., 2016). This may be due to repetitive short-duration exercises which induced changes in glycolytic enzymes, muscle buffering, and ion regulation and led to improved anaerobic capacity (Dawson, 2012) rather than aerobic performance.

Based on two articles, we cannot get more information from these two variables, thus, in the future, more studies evaluating the effects of PT on reaction time and aerobic endurance are required to reach more reliable conclusions.

### Limitations

This systematic review can be regarded as having some limitations, which should be taken into account. The first limitation is that only 12 publications were analyzed, and there were insufficient RCTs (*n* = 5) included in this study. Secondly, there is no study specifically analyzing female tennis players in this review, which limits our comprehension of the overall effectiveness of PT in tennis players. This presents a crucial gap in the study, and should be addressed in further studies. Thirdly, three studies were excluded from the current meta-analysis because they lacked control group data or did not offer enough. In addition, several outcomes (such as serve accuracy, upper extremity power and strength) were excluded from our meta-analysis, since there was an insufficient number of studies involved. Fourthly, several studies did not give a comprehensive description of the training program; for example, three of the included publications did not include details regarding the training protocol. Fifthly, the majority of the studies included in this review had small sample sizes (8–30 people per EG), and only one research disclosed the sample size calculation technique (Mohanta et al., 2019), meaning statistically significant results may not reflect the actual effect. As demonstrated by GRADE, the certainty of the evidence ranged from very low to moderate for most of the study outcomes, lowering confidence in the reported estimates.

## Conclusion

To summarize, this systematic review and meta-analysis demonstrated that PT implicates muscle stimulus that induces neuro-mechanical adaptations, which could increase force production, thereby improving tennis performance, including maximal serve velocity and physical performance components (i.e., sprint speed, lower extremity muscle power, agility). There was no definitive evidence that PT changed serve accuracy, upper extremity power and strength, reaction time, and aerobic endurance. According to GRADE, the certainty of evidence is very low to moderate in studies. Future high-quality evidence is needed to demonstrate the effects of PT on skill and physical performance in tennis players.

## Practical application

The findings of this review have practical implications for athletic trainers, coaches, and athletes. PT might be advised as a training method to boost maximal serve velocity or different parameters of physical performance in healthy tennis players. These factors are essential for enhancing the competitive level of tennis players (Lambrich and Muehlbauer, 2022). Moreover, one of the primary advantages of this type of training is that it requires only inexpensive equipment (e.g., jump box, medicine ball), so it can be readily incorporated into the daily training regimen (Fernandez-Fernandez et al., 2013). Regarding the characteristics of effective PT interventions, it appears that a training frequency of two to three sessions per week for six to nine weeks is a sufficient stimulus to obtain improvement. However, due to the limited number of high-quality research conducted on the topic, specific recommendations cannot be made. More researchers are encouraged to explore the effects of PT on the athletic performance of tennis players.

## Data Availability

The original contributions presented in the study are included in the article/Supplementary Material, further inquiries can be directed to the corresponding authors.
