# Supplementary material for: Effects of plyometric training on skill and physical performance in healthy tennis players: A systematic review and meta-analysis
Source: Front Physiol. 2022 Nov 24;13:1024418. doi: 10.3389/fphys.2022.1024418 (PMC9729950; doi:10.3389/fphys.2022.1024418)
Supplement: Supplementary file 1 [file DataSheet1.ZIP › Appendix B.docx]

| **Appendix 2** | **The data used for meta-analyses** | | | |  | | | | | |  | | |
| --- | --- | --- | --- | --- | --- | --- | --- | --- | --- | --- | --- | --- | --- |
| **Reference** | **Test** | **Experimental (pre-test)** | | | **Experimental (pos-test)** | | | **Control (pre-test)** | | | **Control (pos-test)** | | |
|  |  | **Mean** | **SD** | **n** | **Mean** | **SD** | **n** | **Mean** | **SD** | **n** | **Mean** | **SD** | **n** |
| Salanikidis and Zafeiridis, 2008 a | Power (CMJ; cm) | 13.6 | 4.4 | 16 | 18.3 | 6.1 | 16 | 14.9 | 2.6 | 8 | 14.7 | 2.8 | 8 |
|  | Strength (Fmax; N) | 1747 | 526 | 16 | 1886 | 580 | 16 | 1716 | 442 | 8 | 1654 | 409 | 8 |
|  | Sprint (12-m forward sprint; s) | 2.47 | 0.2 | 16 | 2.44 | 0.22 | 16 | 2.39 | 0.13 | 8 | 2.41 | 0.11 | 8 |
| Salanikidis and Zafeiridis, 2008 b | Power (CMJ; cm) | 13.8 | 5.5 | 16 | 16.9 | 4.8 | 16 | 14.9 | 2.6 | 8 | 14.7 | 2.8 | 8 |
|  | Strength (Fmax; N) | 1732 | 838 | 16 | 1874 | 883 | 16 | 1716 | 442 | 8 | 1654 | 409 | 8 |
|  | Sprint (12-m forward sprint; s) | 2.47 | 0.19 | 16 | 2.41 | 0.19 | 16 | 2.39 | 0.13 | 8 | 2.41 | 0.11 | 8 |
| Behringer et al. 2013 | Strength (10RM Leg press; kg) | 20.4 | 7.6 | 12 | 26.3 | 11.1 | 12 | 20.6 | 7.7 | 12 | 21.5 | 7.2 | 12 |
|  | Maximal serve velocity (km/h) | 127.4 | 12.5 | 12 | 131.2 | 12.3 | 12 | 115.1 | 19.1 | 12 | 109 | 18 | 12 |
| ÖLÇÜCÜ et al. 2013 | Maximal serve velocity (km/h) | 123.8 | 10.8 | 20 | 144.8 | 10.6 | 20 | 117 | 10.3 | 20 | 128.4 | 9.9 | 20 |
|  | Strength (knee joint;N) | 168.2 | 52.6 | 20 | 192.5 | 64 | 20 | 164.7 | 51.4 | 20 | 172.5 | 52.4 | 20 |
| Fernandez-Fernandez et al. 2015 | Power (CMJ; cm) | 41.7 | 2.1 | 8 | 42.6 | 1.8 | 8 | 40.5 | 1 | 8 | 40.39 | 0.1 | 8 |
|  | Sprint (10 m sprint; s) | 1.84 | 0.1 | 8 | 1.79 | 0.1 | 8 | 1.86 | 0.1 | 8 | 1.88 | 0.1 | 8 |
| Rathore et al. 2016 | Agility (Illinois test; s) | 18.71 | 0.69 | 20 | 16.58 | 0.44 | 20 | 18.55 | 0.71 | 20 | 17.13 | 0.88 | 20 |
| Fernandez-Fernandez et al. 2016 | Power (CMJ; cm) | 31.1 | 4.3 | 24 | 32 | 4.1 | 24 | 30.3 | 4.3 | 27 | 30.9 | 4 | 27 |
|  | Sprint (10 m sprint; s) | 2.01 | 0.1 | 24 | 1.93 | 0.1 | 24 | 2 | 0.1 | 27 | 1.99 | 0.1 | 27 |
|  | Agility (5-0-5 test; s) | 2.95 | 0.2 | 24 | 2.86 | 0.2 | 24 | 2.93 | 0.1 | 27 | 2.92 | 0.1 | 27 |
|  | Maximal serve velocity (km/h) | 138.6 | 8.2 | 24 | 147.3 | 14 | 24 | 140.1 | 9.4 | 27 | 141 | 7.8 | 27 |
| Lakshmikanth et al. 2018 | Agility (Illinois test; s) | 24.78 | 1.88 | 15 | 23.2 | 1.41 | 15 | 23.67 | 1.03 | 15 | 20.52 | 1.65 | 15 |
| Ziagkas et al. 2019 | Agility (Spider test) | 16.4 | 2.28 | 12 | 11.7 | 2.43 | 12 | 16.6 | 2.18 | 12 | 15.2 | 2.22 | 12 |

Note: a, plyometric group 1; b, plyometric group 2; CMJ, countermovement jump; Fmax, maximum isometric force (leg)
