# Supplementary material for: Effects of plyometric training on skill and physical performance in healthy tennis players: A systematic review and meta-analysis
Source: Front Physiol. 2022 Nov 24;13:1024418. doi: 10.3389/fphys.2022.1024418 (PMC9729950; doi:10.3389/fphys.2022.1024418)
Supplement: Supplementary file 1 [file DataSheet1.ZIP › Appendix C.docx]

**Appendix C:** GRADE Assessment for RCTs

| **No. of Studies (total participants)** | **Design** | **Study Limitation** | **Inconsistency** | **Indirectness** | **Imprecision** | **Publication Bias** | **Certainty**  **(overall score)** |
| --- | --- | --- | --- | --- | --- | --- | --- |
| **Outcome:** Serve velocity | | | | | | | |
| Gelen et al., 2012  Behringer et al., 2013  (n=62) | Behringer et al., 2013; RCT  Gelen et al., 2012  : within-subject, randomized, repeated-measures design | Downgraded one level for serious limitation: Gelen et al (2013) had some concerns due to deviation from the intended intervention , no clear description of the randomization process, and select reported result. | **Differences in interventions:**  Behringer et al., 2013 conducts a 8-week PT whereas Gelen et al., 2012 was a acute effects of study. | Not Downgraded. | Downgraded one level for serious imprecision: small sample size, they did not reported CI | None detected | 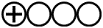 **Very low** |
| **Outcome: Serve accuracy** | | | | | | | |
| Behringer et al., 2013  (n=36) | RCT | No downgrading | Only one study meaning inconsistency is not applicable. | No downgrading | Downgraded one level due to serious imprecision: The evidence for this outcome is based on one study which reported no change in serve accuracy (P > 0.05). And this study only included 10 participants in the PT group, which may impact the statistical power of results | None detected | 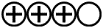 **Moderate** |
| **Outcome: Sprint speed** | | | | | | | |
| Salanikidis and Zafeiridis, 2008  (n=64) | RCT | Downgraded one level for serious limitation: this study had some concerns due to no clear description of the randomization process. | Only one study meaning inconsistency is not applicable. | Not Downgraded. | Downgraded one level for serious imprecision: This outcome is based on just one study which reported no effect in some distance of sprint. This study included 16 participants in the PT group, which may impact the statistical power of results. | None detected | 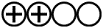 **Low** |
| **Outcome:** **Upper and lower extremity power** | | | | | | | |
| Salanikidis and Zafeiridis, 2008  (n=64) | RCT | Downgraded one level for serious limitation: this study had some concerns due to no clear description of the randomization process. | Only one study meaning inconsistency is not applicable. | Not Downgraded. | Downgraded one level due to serious imprecision: The evidence for this outcome is based on one study which reported improvement in upper and lower extremity power (P < 0.05). However, this study only included 16 participants in the PT group, which may impact the statistical power of results. | None detected | 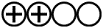 **Low** |
| **Outcome:** upper and lower extremity strength | | | | | | | |
| Behringer et al., 2013  Salanikidis and Zafeiridis, 2008  (n=100) | RCTs | Downgraded one level for serious limitation: Salanikidis and Zafeiridis, 2008 had some concerns due to no clear description of the randomization process. | **Differences in interventions:**  Behringer et al., 2013 conducts a 8-week PT whereas Salanikidis and Zafeiridis, 2008 conducts a 9 week of PT | Not Downgraded | Downgraded one level for serious imprecision: small sample size, which may impact the statistical power of results. | None detected | 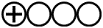 **Very low** |
| **Outcome: Agility** | | | | | | | |
| Rathore, 2016  Ziagkas et al., 2019  (n= 84) | RCTs | Downgraded one level for serious limitation: these studies had some concerns in risk of bias assessment | Not downgraded | Not downgraded | Downgraded one level for serious imprecision: small sample size, the total number of participants across studies for this outcome was 32, which may impact the statistical power of results. | None detected | 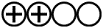 **Low** |
| **Outcome: reaction time** |  |  |  |  |  |  |  |
| Salanikidis and Zafeiridis, 2008 | RCT | Downgraded one level for serious limitation: this study had some concerns in RBO2 assessment | Only one study meaning inconsistency is not applicable. | Not downgraded | Downgraded one level for serious imprecision: small sample size, this study only included 16 participants in the PT group, which may impact the statistical power of results. | None detected | 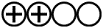 **Low** |
| **Outcome: aerobic endurance** |  |  |  |  |  |  |  |
| **No RCT trial** | － | － | － | － | － | － | － |

**Appendix 5:** GRADE assessment for NRSIs

| **No. of Studies (total participants)** | **Design** | **Study Limitations** | | **Inconsistency** | **Indirectness** | | **Imprecision** | | **Publication Bias** | **Certainty**  **(overall score)** |
| --- | --- | --- | --- | --- | --- | --- | --- | --- | --- | --- |
| **Outcome: maximal serve velocity** | | | | | | | | | | |
| ÖLÇÜCÜ et al., 2013  Fernandez-Fernandez et al., 2016  Fernandez-Fernandez et al., 2018  (n=116) | Non-RCTs | **Downgraded** for limitations in design, some concern risk of bias in ROB-2 assessment. | | **Do not downgrade** | **Downgraded one lelve by PT combined with other type of training (** Fernandez-Fernandez et al., 2018). | | **Serious imprecision:** Small sample size, no sample size calculation, no study reported large sample size effects. | | None detected | 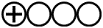 **Very low** |
| **Outcome: Serve accuracy** | | | | | | | | | | |
| Fernandez-Fernandez et al., 2016  (n=60) | Non-RCT | **Downgraded** for limitations in design | | Only one study meaning it is not possible to judge for inconsistency.  **Do not downgrade** | No indirectness | | **Serious imprecision:** Small sample size ), no sample size calculation | | None detected | 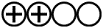 **Low** |
| **Outcome:** **Sprint speed** | | | | | | | | | | |
| Fernandez-Fernandez et al., 2015  Fernandez-Fernandez et al., 2016  Fernandez-Fernandez et al., 2018  Mohanta et al., 2019  (n=132) | Non-RCTs | **Downgraded** for limitations in design | | **Direction and magnitude of results across studies** were different.  Fernandez-Fernandez et al., 2016 and Mohanta et al., 2019  showed increase 5m-20m and 50m respectively, however, Fernandez-Fernandez et al., 2015 did not change (20m, 30m) and Fernandez-Fernandez et al., 2018 showed decrease on 20m | **Downgraded one lelve by PT combined with other type of training (**Fernandez-Fernandez et al., 2015 and  Fernandez-Fernandez et al., 2018). | | **Serious imprecision:** Small sample size, no sample size calculation | | None detected | 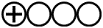 **Very low** |
| **Outcome:** **Upper and lower power** | | | | | | | | | | |
| Fernandez-Fernandez et al., 2015;  Fernandez-Fernandez et al., 2016;  Fernandez-Fernandez et al., 2018  (n=92) | Non-RCTs | **Downgraded** for limitations in design | **Do not downgrade** | | | **Downgraded one lelve by PT combined with other type of training (**Fernandez-Fernandez et al., 2015 and  Fernandez-Fernandez et al., 2018). | | **Serious imprecision:** Small sample size, no sample size calculation | None detected | 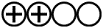 **Low** |
| **Outcome:** **upper and lower strength** | | | | | | | | | | |
| ÖLÇÜCÜ et al., 2013  Mohanta et al., 2019 | Non-RCTs | **Downgraded** for limitations in design | **Do not downgrade** | | | **Do not downgrade** | | **Serious imprecision:** Small sample size, no sample size calculation (ÖLÇÜCÜ et al., 2013) | None detected | 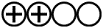 **Low** |
| **Outcome: Agility** |  |  |  | | |  | |  |  |  |
| Fernandez-Fernandez et al., 2016  Fernandez-Fernandez et al., 2018  Mohanta et al., 2019; Hotwani, 2021  Lakshmikanth et al., 2018 | Non-RCTs | **Downgraded** for limitations in design  **Upgraded** one level for Fernandez-Fernandez et al., 2016 excluded injuries participants | **Differences in interventions:**  Fernandez-Fernandez et al., 2016, Fernandez-Fernandez et al., 2018, Hotwani, 2021 and  Lakshmikanth et al., 2018 conducts an 8-week PT whereas Mohanta et al., 2019 conducts a 3-week PT. | | | **Downgraded one lelve by PT combined with other type of training (**Fernandez-Fernandez et al., 2015, 2018 and Hotwani, 2021). | | **Serious imprecision:** Small sample size, no sample size calculation | None detected | 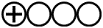 **Very low** |
| **Outcome: Reaction time** |  |  |  | | |  | |  |  |  |
| No study | － | － | － | | | － | | － | － | － |
| **Outcome: Aerobic endurance** |  |  |  | | |  | |  |  |  |
| Fernandez-Fernandez et al., 2015  (n=16) | Non-RCT | **Downgraded** for limitations in design | Only one study meaning it is not possible to judge for inconsistency.  **Do not downgrade** | | | **Downgraded** one lelve by PT combined with other type of training | | **Serious imprecision:** Small sample size, no sample size calculation | None detected | 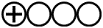 **Very low** |
