# Supplementary material for: Effects of plyometric training on skill and physical performance in healthy tennis players: A systematic review and meta-analysis
Source: Front Physiol. 2022 Nov 24;13:1024418. doi: 10.3389/fphys.2022.1024418 (PMC9729950; doi:10.3389/fphys.2022.1024418)
Supplement: Supplementary file 1 [file DataSheet1.ZIP › Appendix D.docx]

| **Appendix D \| Plyometric Training Protocols.** | | | | | | | | | |
| --- | --- | --- | --- | --- | --- | --- | --- | --- | --- |
| **Study** | **Intensity** | **Exercises** | **Combined** | **RBS (s)** | **RBR (s)** | **RBTS (h)** | **Replace** | **TP** | **PO** |
| Salanikidis and Zafeiridis (2008) | NR | LBPT= (all single-leg) hops,hopping on stairs (20–40 cm), ‘‘kangaroo’’ jumps and knee lifting to the chest, ‘‘jump and reach’’ in place, drop jumps (20-40 cm), zig-zag lateral hops over the sideline;  2 sets × 6–8 repetitions each | No | 180-240 | 60-120 | NR | NR | NR | NR |
| Gelen et al. (2012) | Low,  Moderate, high* | UBPT=ballistic six exercises using theraband, 1 kg and 3 kg medicine balls;  1 set× 20 repetitions each | No | NR | 60 | NR | No | IS | I |
| Behringer et al. (2013) | NR | UBPT=push-ups with and without clapping hands, medicine ball chest pass, two-hand overhead throw with and without upper body rotation;  LBPT: rope skipping/lateral barrier hop (single-and double-leg), box hopping (clock- and counter-clockwise; single- and double-leg), countermovement jump, counter movement jump to box, cycled split squat jump;  3-4sets × 10-15 repetitions each | No | 20-60 | 0-1 | 55-78 | No | NR | comb |
| ÖLÇÜCÜ et al. (2013) | Max | 2 sets ×12 repetitions each | No | NR | 60-120 | NR | Yes | NR | NR |
| Fernandez-Fernandez et al. (2015) | Max | LBPT= box jumps, plyometric jumps (hurdles), calf jumps,ladder drills, resisted movements, multilateral hops (hurdles);  3-4 sets ×12–15 repetitions each session | Sprint training | 180 | 45 | 48 | No | IS | T |
| Fernandez-Fernandez et al. (2016) | Max | UBPT= (2-kg medicine ball) chest throw, overhead throw/slam, close- stance throw, open/close-stance throw, 2-hand overhead throw with rotation, push-ups (clapping hands);  LBPT= 2/1-foot ankle hop forward/lateral, 2/1-leg box hopping, 2/1-leg multidirectional hurdle jumps; 2/1-leg zigzag over lines, lateral bounds + stabilization, CMJ;  2 -4 sets ×10 -15 repetitions each session | No | 15-90 | NR | NR | Yes | IS | comb |
| Rathore (2016) | NR | Not described | No | NR | NR | NR | NR | NR | NR |
| Fernandez-Fernandez et al. (2018) | Max | UBPT= medicine ball throws (2 kg);  LBPT= countermovement jump, drop landings (20 cm), multi-jumps (20-60 cm hurdles), drop jumps (20-40 cm), ankle jumps, line jumps, lateral bounds with stabilization;  2-3 sets ×4–10 repetitions each | Acceleration/deceleration/COD drills | 60 | 15-20 | NR | No | NR | V.T |
| Lakshmikanth et al. 2018 | Max | LBPT= side to side ankle hops, standing jump and reach, front/diagonal/lateral cone hops, lateral jump over barrier, double leg hops, standing long jump with and without lateral sprint, lateral jump single leg cone hops with 180 degree than, single leg bounding, hexagon drill, cone hops with change of direction sprints. | No | NR | NR | NR | No | NR | NR |
| Ziagkas et al. (2019) | Max | 2–4 sets and 10–15 repetitions each | No | 15-90 | 15-90 | NR | Yes | NR | NR |
| Mohanta et al. (2019) | Max | UBPT= (medicine ball) chest throw; overhead throw/ slam; close- stance throw, open/close-stance throw; 2-hand overhead throw with rotation, push-ups (clapping hands);  LBPT= 2/1-foot ankle hop forward/lateral, 2/1-leg box hopping, 2/1-leg multidirectional hurdle jumps; 2/1-leg zigzag over lines, lateral bounds + stabilization, countermovement jump;  2-4 sets ×10-15 repetitions each | NR | 15 | 90 | NR | No | NR | Com |
| Hotwani (2021) | NR | LBPT= single leg hopping, hurdle jumping, zig-zag hops, zig-zag runs (forward and lateral), 3-5 cone drills, Zigzag runs with zig-zag hops, tuck jumps and single leg hop, carioca four corner  3 sets×10 repetitions each | Sprint  training | 600 | 180 | NR | NR | NR | NR |

UBPT, upper body plyometric training; LBPT,lower body plyometric training; COD, change of direction; TP, training period;IS, in- season;NR, not reported; PO, progressive overload, in the form ofeither volume (i.e., V), intensity (i.e., I), type of drill (i.e., T), or a combination ofthese (Comb);RBR, rest time between repetitions; RBS, rest time between sets; RBTS, rest between training sessions;Max, maximal, involving either maximal effort to achieve maximal height, distance, reactive strength index, velocity (time contact or fast stretch-shortening cycle), or another marker of intensity, For the studies marked with an *, the intensity was reported only qualitatively; replace, replacement of a portion of the regular tennis drills with plyometric drills
