# Supplementary material for: Effects of plyometric training on skill and physical performance in healthy tennis players: A systematic review and meta-analysis
Source: Front Physiol. 2022 Nov 24;13:1024418. doi: 10.3389/fphys.2022.1024418 (PMC9729950; doi:10.3389/fphys.2022.1024418)
Supplement: Supplementary file 1 [file DataSheet1.ZIP › Appendix E.docx]

**Appendix E Forest plots**

**FIGURE 1 |** Forest plot of changes in maximal serve velocity performance, in participants that completed a plyometric training program compared to participants allocated as controls. Values shown are effect sizes (Hedges’s g) with 95% confidence intervals (CI). The size of the plotted squares reflects the statistical weight of each study. Note: O = ÖLÇÜCÜ et al

**FIGURE 2 |** Forest plot of changes in sprint speed performance, in participants that completed a plyometric training program compared to participants allocated as controls. Values shown are effect sizes (Hedges’s g) with 95% confidence intervals (CI). The size of the plotted squares reflects the statistical weight of each study. Notes: a = plyometric group 1; b = plyometric group 2

**FIGURE 3 |** Forest plot of changes in lower extremity power performance, in participants that completed a plyometric training program compared to participants allocated as controls. Values shown are effect sizes (Hedges’s g) with 95% confidence intervals (CI). The size of the plotted squares reflects the statistical weight of each study. Notes: a = plyometric group 1; b = plyometric group 2

**FIGURE 4 |** Forest plot of changes in lower extremity muscle strength, in participants that completed a plyometric training program compared to participants allocated as controls. Values shown are effect sizes (Hedges’s g) with 95% confidence intervals (CI). The size of the plotted squares reflects the statistical weight of each study. Notes: a = plyometric group 1; b = plyometric group 2; O = ÖLÇÜCÜ et al

**FIGURE 5 |** Forest plot of changes in agility, in participants that completed a plyometric training program compared to participants allocated as controls. Values shown are effect sizes (Hedges’s g) with 95% confidence intervals (CI). The size of the plotted squares reflects the statistical weight of each study.
