# Supplementary material for: Effects of plyometric training on skill and physical performance in healthy tennis players: A systematic review and meta-analysis
Source: Front Physiol. 2022 Nov 24;13:1024418. doi: 10.3389/fphys.2022.1024418 (PMC9729950; doi:10.3389/fphys.2022.1024418)
Supplement: Supplementary file 1 [file DataSheet1.ZIP › Appendix A.docx]

**Supplementary Appendix A**

**Detailed search strategy**

**Search Date: 30 May 2022**

|  | (P) Tennis players (I) Plyometric training | Results |
| --- | --- | --- |
| Pubmed  (1946 – May 2022) | ("plyometric training" [Title/Abstract] OR "plyometric exercise*" [Title/Abstract] OR "plyometric drill*"[Title/Abstract] OR "plyometr*"  [Title/Abstract] OR "ballistic training"[Title/Abstract] OR "explosive"  [Title/Abstract] OR "force-velocity"[Title/Abstract] OR "stretch-shortening cycle"[Title/Abstract] OR "stretch-shortening exercise" [Title/Abstract] OR "complex training"[Title/Abstract] OR "jump training"[Title/Abstract]) AND ("tennis"[Title/Abstract] OR "tennis player*"[Title/Abstract] OR "tennis athlete*"[Title/Abstract]) | 52 |
| Web of Science  (1945 – May 2022) | (AB=("plyometric training" OR "plyometric exercise*" OR "plyometric drill*" OR "plyometr*" OR "ballistic six" OR "ballistic training" OR "explosive" OR "force-velocity" OR "stretch-shortening cycle" OR "stretch-shortening exercise" OR "complex training" OR "jump training")) AND AB=("tennis" OR "tennis player*" OR "tennis athlete*") | 116 |
| EBSCOhost  (1984 - May 2022)  (SPORTDicus) | AB ("plyometric training" OR "plyometric exercise*" OR "plyometric drill*" OR "plyometr*" OR "ballistic six" OR "ballistic training" OR "explosive" OR "force-velocity" OR "stretch-shortening cycle" OR "stretch-shortening exercise" OR "complex training" OR "jump training") AND AB ("tennis" OR "tennis player*" OR "tennis athlete*") | 98 |
| Scopus  (2004 – May 2022) | (TITLE-ABS-KEY("plyometric training"OR "plyometric exercise*"OR "plyometric drill*"  OR  "plyometr*"  OR  "ballistic six"  OR  "ballistic training"  OR  "explosive"  OR  "force velocity"  OR "stretch-shortening cycle"  OR  "stretch-shortening exercise" OR  "complex training"OR"jump training") AND TITLE-ABS-KEY("tennis"OR  "tennis player*"  OR  "tennis athlete*" )) | 95 |
| Total |  | 361 |
